# The relation of energy cost of walking with gait deviation, asymmetry, and lower limb muscle co-activation in children with cerebral palsy: a retrospective cross-sectional study

**DOI:** 10.1186/s12891-023-06223-1

**Published:** 2023-02-09

**Authors:** Yngvild Gagnat, Siri Merete Brændvik, Inge Ringheim, Karin Roeleveld

**Affiliations:** 1 Clinic for Orthopaedics, Rheumatology and Skin Diseases, Orthopaedic Research Center, St. Olavs University Hospital, Trondheim, Norway; 2 Department of Neuromedicine and Movement Science, Faculty of Medicine and Health Sciences, Norwegian University of Science and Technology, Trondheim, Norway; 3 Clinical Services, St. Olavs University Hospital, Trondheim, Norway; 4 Division of Physical Medicine and Rehabilitation, Vestfold Hospital Trust, Stavern, Norway

**Keywords:** Energy cost, Cerebral palsy, Gait deviation, Gait asymmetry, Muscle co-activation

## Abstract

**Background:**

Compared to typically developing children, children with cerebral palsy (CP) have increased energy expenditure during walking, limiting activity and participation. Insight into whether the also deviating and more asymmetric gait with increased muscle co-activation contributes to this increased energy expenditure is important for clinical decision making. The aim of this study was to investigate the relation between energy cost of walking with gait deviation, asymmetry, and muscle co-activation in children with CP.

**Methods:**

Forty ambulant children with CP, with Gross Motor Function Classification System (GMFCS) level I (*N* = 35) and II (*N* = 5), aged between 5-17y, were tested at one or two occasions with 24 weeks in between, resulting in 71 observations. Gross energy cost (J/kg/m) was measured during a 5-min walk test at self-selected speed. From a 3-dimensional gait analyses, kinematic variables and electromyography were extracted to calculate the gait deviation index (GDI) and co-activation index. The relation between energy cost and GDI, GDI asymmetry, and co-activation index of the lower limb muscles was evaluated through mixed model analyses. Height was included to control for growth-related variation.

**Results:**

Gait deviation and height combined explained about 40% of the variance in gross energy cost. No significant contribution was found for gait asymmetry or co-activation index.

**Conclusions:**

This cross-sectional study indicates that increased gait deviation contributes to increased energy cost of walking in children with GMFCS level I and II.

## Highlights

• Growth-related subject characteristics are strongly related to energy cost.

• Increased gait deviation is related to increased energy cost.

• Gait asymmetry and muscle co-activation are not related to energy cost.

## Background

Increased energy expenditure during walking may lead to activity limitations and reduced participation in daily life [1]. Cerebral palsy (CP) is the most common cause of neuromuscular disability in childhood and ambulant children with CP have increased energy expenditure during walking compared to typically developing children [2–4]. Therefore, an often used treatment goal for these children with CP is to make walking less energy demanding. Knowledge about factors affecting walking energy expenditure in this young patient group will thus aid clinical decision making and planning of a suitable course of treatment to achieve this goal.

Children with CP have impaired motor control and balance, and reduced muscle strength [5]. These features impair gross motor function in general and gait in particular as reflected in the Gross Motor Function Classification System (GMFCS) [6]. Moreover, the ambulant children with CP (GMFCS I to III), have an increasing energy expenditure during walking with decreasing motor function [4, 7, 8]. The gait pattern in children with CP is characterized by deviations in joint kinematics that may be present at several levels (pelvis, hip, knee, ankle, foot) and in all three movement planes [9]. Increased walking energy expenditure has been associated with kinematic deviations [10].

Another gait characteristic affected by CP is asymmetry, reflected both in joint kinematics and spatiotemporal gait parameters [11, 12]. An asymmetric gait pattern is reported to be more energy demanding than symmetric walking in the normal population and in stroke patients [13–15]. However, there is still uncertainty how an asymmetric gait pattern affects energy expenditure during walking in children with CP.

Moreover, the excessive lower limb muscle co-activation reported in children with CP during walking [16–19] may also play a role in increased energy expenditure. Muscle co-activation may be defined as concurrent activity of agonist and antagonist muscles crossing the same joint [20]. It has functional benefits by increasing joint stabilization, co-ordination, and the ability to perform challenging tasks, such as walking [20]. On the other hand, excessive and prolonged co-activation may deteriorate movement and increase energy expenditure by decreasing flexibility and adaptability and increase the total muscle force involved in generating joint moments. However, previous research on the relation between walking energy expenditure and lower limb muscle co-activation in children with CP have methodological limitations and present contradictory findings. Evaluated through oxygen uptake, leg and thigh muscle co-activation have shown to be positively related [18] or not related [21] to walking energy expenditure. While evaluated through heart rate changes, co-activation in the thigh muscles was negatively related to walking energy expenditure [22]. Although heart rate is closely related to oxygen uptake and oxygen uptake to aerobic energy expenditure, energy expenditure is also dependent on the type of fuel utilised and possible anaerobic processes [23].

To what extent deviations in gait, asymmetry, and muscle co-activation affect walking energy expenditure in children with CP is still deficient and ambiguous, although knowledge on this relation is useful for clinical reasoning. Therefore, the aim of this paper was to investigate energy cost of walking in ambulant children with CP in relation to gait deviation, gait asymmetry, and co-activation of lower limb muscles.

## Methods

This is a retrospective cross-sectional study, based on parts of data from an ongoing randomized control trial [24]. The protocol used for those data sets has been published previously [17, 23].

### Participants

Forty ambulant children diagnosed with unilateral or bilateral spastic CP, classified with GMFCS level I and II were included in this study. Thirty-one children were tested twice with 24 weeks in between, and nine children were only tested once, resulting in a total of 71 observations. Characteristics of the children are presented in Table 1. The children had not been treated with botulinum toxin-A in the lower limb muscles in the preceding three months or undergone surgery in the lower legs preceding 24 months prior to both testing occasions.

**Table 1:** Characteristics of the included observations and descriptive data. Presented as mean ± standard deviation (SD) and 95% confidence interval (CI) and/or number of observations (N) derived from 40 participants

Variable	Mean ± SD	(95% CI)	N
Age (y)	9.6 ± 3.2	(8.8 – 10.3)	71
Gender (girls/boys)			33/38
Height (cm)	136.4 ± 19.1	(131.9 – 141.0)	71
Bodyweight (kg)	33.5 ± 14.9	(30.0 – 37.1)	71
BSA (m^2^)	1.12 ± 0.32	(1.04 – 1.19)	71
Unilateral/Bilateral			57/14
GMFCS level I/II			62/9
Normalised walking speed 5MWT	0.42 ± 0.06	(0.41 – 0.44)	71
Energy cost (J/kg/m)	5.14 ± 1.56	(4.77 – 5.51)	71
GDI	76.9 ± 8.2	(74.9 – 78.8)	71
GDI asymmetry	13.3 ± 9.6	(11.0 – 15.5)	71
CoA index	38.7 ± 11.4	(35.9 – 41.5)	66

*BSA* Body surface area, *GMFCS* Gross motor function classification system, *5MWT* 5-min walk test, *GDI* Gait deviation index, *CoA* Co-activation

### Procedure and equipment

Characteristics of the children were recorded prior to testing, including age, height, and bodyweight.

Energy expenditure and distance walked were measured during a 5-min walk test (5MWT) [3] performed on a 45-m marked pathway. The children wore shoes and walked at self-selected, comfortable walking speed. An indirect calorimeter, Metamax, version II or IIIb (Cortex Biophysik GmbH, Leipzig, Germany) carried on the back was used to measure oxygen uptake (VO_2_) and carbon dioxide production (VCO_2_). Calibration of the calorimeter prior to testing was done according to the manufacturer’s instructions. The children wore a facemask placed over mouth and nose, carefully inspected for leakage.

In addition, a 3-dimensional gait analysis (3DGA) with simultaneously surface electromyography (sEMG) recordings was conducted during barefoot walking at self-selected, comfortable walking speed. The children conducted a minimum of three trials. At least three complete gait cycles for each leg were extracted for analyses. For 51 observations, 3DGA was conducted using Vicon Motion System (Ltd, Oxford, UK) and for 20 observations using Qualisys Motion Capture System (Qualisys AB, Gothenburg, Sweden). The sEMG recordings were conducted using Myon wireless sEMG system (Myon AG, Switzerland) for 46 observations, using Cometa MiniWave sEMG system (Cometa Srl, Bareggio, Italy) for 13 observations, and using DelSys Trigno Avanti wireless sEMG system (DelSys Inc, Natick, MA, USA) for seven observations. At least seven cameras with a sampling frequency of at least 150 Hz, and two AMTI force plates (Watertown, USA), with a sampling frequency of at least 1000 Hz were positioned along a 7-to-8 m walkway. Reflective markers (16, 20 or 28) were placed on anatomical landmarks on the lower limbs, according to the Vicon Plug-in-Gait model [25], the IOR [26] or the Qualisys CAST [27] lower body marker set, respectively. For the sEMG measurements, preparation of the skin and electrode placement were done according to the SENIAM (Surface Electromyography for the Non-Invasive Assessment of Muscles) guidelines [28]. The sEMG was recorded bilaterally of m. tibialis anterior (TA), m. soleus (SOL), m. gastrocnemius medialis (GM), m. rectus femoris (RF) and m. hamstring medialis (HM). sEMG was amplified by a 1000 gain and sampled at a frequency of at least 1000 Hz.

### Data analysis

To investigate growth-related variation on energy cost [7], body surface area (BSA, m^2^) was calculated using Eq. 1 [29]:

1 $${BSA\;(m}^{2})= \sqrt{\frac{height\;\left(cm\right)\;\times\;bodyweight\;\left(kg\right)}{3600}}$$

BSA = Body surface area

### Energy expenditure

To evaluate energy expenditure, gross energy cost, defined as the energy used per unit of distance walked, was assessed. Energy cost is acknowledged as a precise indicator of walking efficiency in children with CP and can reliably be determined using a 5MWT protocol [3]. Oxygen uptake (VO_2_) and carbon dioxide production (VCO_2_) were averaged over a 1-min steady state period during the last two minutes of the 5MWT. The respiratory exchange ratio (RER) was calculated by dividing VO_2_ by VCO_2_. The mean VO_2_ relative to bodyweight (ml/kg/min), RER and walking speed (m/min) were used to calculate the energy cost (J/kg/m) using Eq. 2 [23]:

2 $$\textit{Energy cost}\;\left(J/kg/m\right)=\frac{\left(4.940\times RER+16.040\right)\times{VO}_2\;(ml/kg/min)}{\textit{walking speed}\;(m/min)}$$

RER = Respiratory exchange ratio, VO_2_ = Oxygen uptake (ml/kg/min).

### Gait deviation and asymmetry

On the Vicon and Qualysis derived kinematic data respectively, Nexus (Oxford Metrics, Oxford, UK) and Qualisys Gait module (Qualisys AB, Gothenburg, Sweden) software were used to define gait cycles, detect events, and calculate spatiotemporal gait parameters. All data from the 3DGA were exported to c3d-files and imported into a customized Matlab program (R2020b, MathWorks, Inc., Natick, MA, USA), written using the Biomechanical Toolkit (Btk Development Core Team, Version 0.3.0), used for consecutive processing and calculations. The gait cycle was normalised to a 100% and kinematic variables were extracted for every percent of the gait cycle. Kinematic variables and spatiotemporal gait parameters were averaged across the included trials to obtain each leg’s mean value.

The gait deviations index (GDI) was calculated from the 3D gait data as described by Schwartz and Rozumalski [30]. Pelvic anterior/posterior tilt, hike/drop, internal/external rotation, hip adduction/abduction, flexion/extension, internal/external rotation, knee flexion/extension, ankle dorsi-/plantarflexion, and foot progression were extracted from every 2% of the normalised gait cycle. GDIs were obtained by comparing individual kinematics to kinematics from 26 typically developing children obtained at our local gait lab. The mean GDI across legs was used for further analyses. A GDI score of 100 or above indicates absence of gait pathology. Every ten points below 100 corresponds to one standard deviation from the average typical gait.

Asymmetry was assessed using the GDI scores of left and right leg, and was calculated using Eq. 3 [31]. Perfect symmetry gives a score of zero, and higher values reflect higher degrees of asymmetry.

3 $$\textit{GDI asymmetry}=(abs(\ln(\textit{GDI left}/\textit{GDI right})))\times100$$

abs = absolute, ln = logarithm, GDI = gait deviation index.

### Muscle co-activation

sEMG data was visually inspected for artefacts and noise. Data of not satisfactory quality, due to e.g. movement artefacts were excluded from analyses regarding muscle activity. The sEMG data was band-pass filtered between 30 and 300 Hz using an 8^th^ order Butterworth filter and sEMG root mean square (RMS) values were computed with a 50 ms moving window. RMS amplitudes were extracted for every percent of the normalised gait cycle, averaged across the included trials, and subsequently normalised to the highest RMS value (peak RMS) throughout the gait cycle. For every percent of the normalised gait cycle the co-activation index was calculated before an overall average of the total gait cycle was obtained (Eq. 4 [32]). The index was further averaged across the included muscle pairs (TA/GM, TA/SOL, and RF/HM).

4 $$\textit{CoA index}=\frac1{100}\sum\nolimits_{p=1}^{p=100}\frac{{RMS}_{low}\left(p\right)}{{RMS}_{high}\left(p\right)}\times\left({RMS}_{low}\left(p\right)+{RMS}_{high}(p)\right)$$

CoA = Co-activation, p = percentage of the normalised gait cycle, RMS_low_ = lowest normalised RMS value of each muscle pair, i.e. the antagonist muscle; RMS_high_ = highest normalised RMS value of each muscle pair, i.e. the agonist muscle.

This co-activation index is thus defined as the ratio between two simultaneously active muscles, an agonist and an antagonist, multiplied by the sum of activity of those muscles. A high co-activation index represents high levels of activity of both the agonist and antagonist muscle, while a low co-activation index represents low levels of activity of both muscles or a high level of activity of one muscle along with a low level of activity of the other muscle [32].

### Statistics

To evaluate the effect of gait deviation, gait asymmetry and lower limb muscle co-activation on variations in energy cost, mixed model analyses were conducted. Subject was set as random factor to account for repeated measures in all models. First, to explore correlations, separate analyses were performed with energy cost (J/kg/m) as dependent variable and each of the growth-related subject characteristics (age, height, bodyweight, and BSA), GDI, GDI asymmetry, and CoA index as independent variables. Subsequently analyses with energy cost (J/kg/m) as dependent variable, and height (the highest correlating growth-related subject characteristic) together with GDI, GDI asymmetry, and CoA index as independent variables. Independent relations in addition to height were obtained for GDI, GDI asymmetry, and CoA index. Normality of residuals was evaluated and confirmed by visual inspection of QQ plots.

Statistical analyses were carried out using SPSS version 27 (IBM Statistics). Significance level was set at *p* < 0.05, and borderline significance are reported where *p* < 0.1.

## Results

Characteristics of the 71 included observations and results from the 5MWT and 3DGA are presented in Table 1. Due to missing data or poor sEMG quality, five observations were completely excluded from the analyses including muscle activity, while 11 observations had one or two muscle pairs removed. The remaining sample consisted of 52 observations with unilateral CP and 14 with bilateral CP, 57 observations with GMFCS level I and nine with GMFCS level II. The mean energy cost was 4.99 J/kg/m (95% CI: 4.63 – 5.3).

Mixed model analysis showed no difference in energy cost, GDI score, GDI asymmetry, nor CoA index between test occasions, observations classified as unilateral and bilateral, or GMFCS level I and II (*p* > 0.26).

**Fig. 1:**
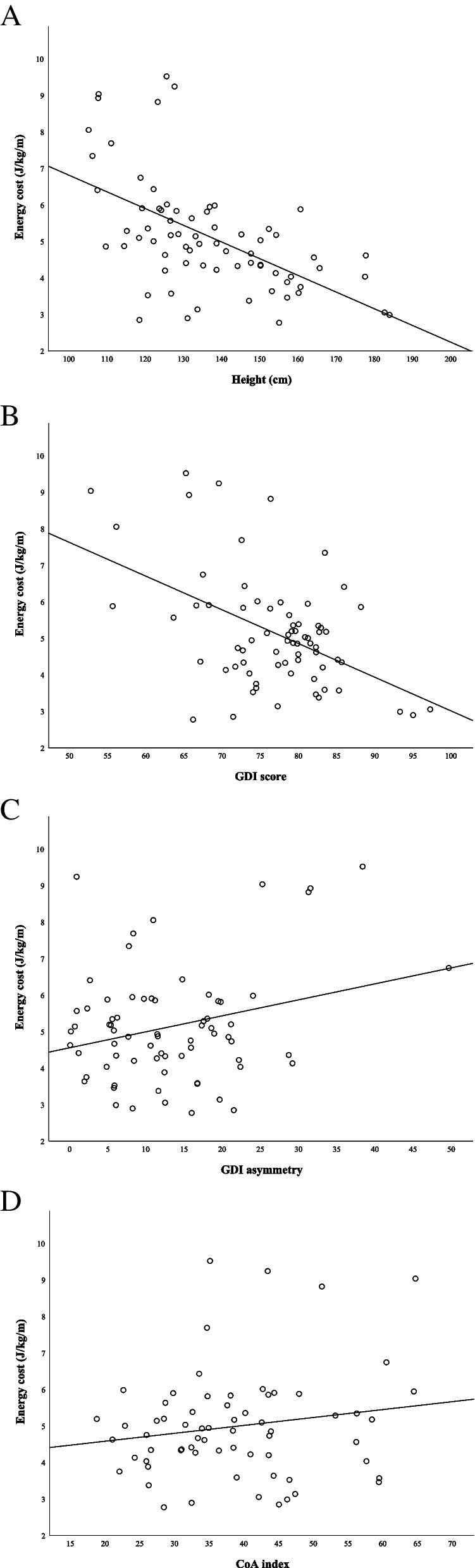
Energy cost (J/kg/m) as a function of height (cm, **A**), gait deviation index (GDI) score (**B**), GDI asymmetry (**C**), and co-activation (CoA) index (**D**)

All growth-related subject characteristics were strongly negatively related to energy cost (explained variance ≥ 26%, *p* < 0.001) with height as the strongest factor (Table 2; Fig. 1A). Decreased GDI score and increased GDI asymmetry were significantly related to increased energy cost (*p* < 0.02; Table 2; Fig. 1B and C). The positive relation between CoA index and energy cost did not reach statistical significance (*p* = 0.2; Table 2; Fig. 1D). For every unit increase in GDI score, energy cost decreased by 0.09 J/kg/m (*p* < 0.001), and for every percentage increase in GDI asymmetry, energy cost increased by 0.04 J/kg/m (*p* = 0.02).

**Table 2:** Results from the mixed model analyses between energy cost (dependent variable) and participant characteristics, gait deviation, gait asymmetry, and muscle co-activation (independent variables). Marginal pseudo-R square (R^2^), slope (B) with its standard errors (SE), significance level (*p*-value), and number of included observations (N) obtained from the 40 participants are presented

	**R**^**2**^	**B (SE)**	***p*-value**	**N**
Age (y)	**0.26**	**-0.25 (0.05)**	** < 0.001**	71
Height (cm)	**0.31**	**-0.05 (0.01)**	** < 0.001**	71
Body weight (kg)	**0.26**	**-0.05 (0.01)**	** < 0.001**	71
BSA (m^2^)	**0.28**	**-2.64 (0.50)**	** < 0.001**	71
GDI	**0.24**	**-0.09 (0.02)**	** < 0.001**	71
GDI asymmetry	**0.07**	**0.04 (0.02)**	**0.02**	71
CoA index	0.03	0.02 (0.02)	0.18	66

*BSA* Body surface area, *GDI* Gait deviation index, *CoA* Co-activation

Mixed model analysis including the highest correlating growth-related subject characteristic, height, in addition to the independent variables, GDI score, GDI asymmetry and CoA index explained 38% of the variance in energy cost (*p* < 0.001) with height and GDI score as significant independent predictors (*p* < 0.002, Table 3). Conducting separate analyses adjusting for height also showed significant relations for GDI score (*p* = 0.002) and borderline significance for GDI asymmetry (*p* = 0.09).

**Table 3:** Results from the mixed model analyses between energy cost (dependent variable) and height, gait deviation, asymmetry, and muscle co-activation (independent variables, left side). Independent relations in addition to height are also included (right side). Marginal pseudo-R square (R^2^), slope (B) with its standard errors (SE), and significance level (*p*-value) obtained from the 40 participants are presented

Variables explaining energy cost (J/kg/m)	R^2^	B (SE)	*p*-value	R^2^	B (SE)	*p*-value
	0.38					
Constant		13.5 (1.88)	< 0.001			
Height (cm)		**-0.03 (0.01)**	** < 0.001**			
GDI		**-0.06 (0.02)**	**0.002**	**0.43**	**-0.07 (0.02)**	** < 0.001**
GDI asymmetry		0.01 (0.02)	0.51	*0.34*	*0.03 (0.02)*	*0.09*
CoA index		-0.01 (0.01)	0.28	0.26	0.02 (0.01)	0.20

*BSA* Body surface area, *GDI* Gait deviation index, *CoA* Co-activation

## Discussion

The aim of this paper was to investigate the relation of energy cost of walking in children with CP with gait deviation, gait asymmetry, and lower limb muscle co-activation. Of these potential contributors to variance in energy cost of walking, only gait deviation showed significant contribution in addition to height in our study sample.

Our results showed that gross energy cost of walking was strongly related to all growth-related subject characteristics. With growth, the energy cost of walking decreased, which is in line with previous studies of children with CP [2, 7]. Although energy cost already is normalised to bodyweight (J/kg/m) it stresses the importance of taking growth into account when evaluating gross energy cost in children with CP.

Deviations in gait pattern, as reflected with the GDI, significantly explained variance in energy cost of walking in our study sample with a partial correlation of approximately 40%. Most of the participants were classified with GMFCS level I (35 out of 40) and a few (five) with GMFCS level II. Our findings thus show that the GDI is sensitive to detect a relation with energy cost even within the least affected children with CP. This is an extension to the knowledge from previous research reporting higher energy cost of walking in higher GMFCS levels and lower motor capacity in standing, reflected by the Gross Motor Function Measure (GMFM) [4, 7]. Noorkoiv and co-workers [10] reported significant relations between increased energy cost and two specific kinematic features, namely reduced knee and hip joint extension. The results were based net energy cost (i.e. energy cost adjusted for rest metabolism) of children with CP with GMFCS levels I-III, and are in agreement with our results.

Neither gait asymmetry nor lower limb muscle co-activation were significantly related to energy cost of walking. Gait asymmetry, reflected as the natural logarithm of the difference between right and left leg’s GDI score, ranged between 0 and 50. The mean score was 13, which is just slightly above the 10% considered to be clinically relevant [33]. This could explain why asymmetry did not explain any significant proportion of the variance, limiting our conclusion to children with CP with GMFCS level I and II. And although there are various measures of asymmetry, they are highly correlated [34]. Thus, choice of measure would probably not have affected our results. Our findings showed co-activation indices between 19 and 65 with an average of 39, similar to other studies of muscle co-activation in children with CP [19]. Our findings are in agreement with the findings of Keefer and co-workers [21], reporting no relation between thigh muscle co-activation and net VO_2_. In contrary, Unnithan and co-workers [18] reported that increased leg and thigh muscle co-activation significantly contributes to increased VO_2_. Various methods have been used to quantify co-activation in the literature and commonly used co-activation indices are based on the ratio between the less and the more activated muscle and the sum of both muscles [18, 21, 35, 36]. A common consequence is that periods of low muscle activity may lead to increased co-activation indices. The co-activation index used in the current study weights the total activation, and may thus provide a better measure of the actual co-activity during dynamic tasks with varying muscle activity [32]. However, regardless of index used, muscle co-activation is reported to be higher in children with CP compared to typically developing peers [16–19].

Although co-activation and gait asymmetry was not related to energy cost, we can not exclude the possibility that reducing co-activation or asymmetry can make walking less energy demaning. In line with this, although gait deviation was related to energy cost, we don’t have information on causality and can not be certain that treatment induced improvements in gait will indeed reduce energy cost of walking and make walking easier. Therefore, longitudinal studies evaluating changes in gait related variables with changes in energy cost should be performed.

There are some considerations regarding the results of this paper. Although both unilateral and bilateral affected children were merged in this study, visual inspection did not indicate systematic differences between the groups in energy cost of walking. Moreover, as gait asymmetry did not significantly contribute to the variance in energy cost, merging seems justified. During the 3DGA, three different models for marker set up were used. Visual inspection of kinematic outcomes showed no differences between the models, and would thus not affect our results.

## Conclusions

This cross-sectional study showed that increased deviation in gait pattern is related to increased energy cost of walking in children with CP with GMFCS level I and II. Additionally, the results of this paper supports that growth must be taken into account when evaluating energy cost of walking in children.

## Data Availability

The datasets generated and/or analysed during the current study are not publicly available due to Norwegian legislation but are available from the corresponding author on reasonable request.

## Abbreviations

BSA: Body surface area
CoA: Co-activation
CP: Cerebral palsy
sEMG: Surface electromyography
GDI: Gait deviation index
GM: Gastrocnemius medialis
GMFCS: Gross motor function classification system
GMFM: Gross motor function measure
HM: Hamstring medialis
RF: Rectus femoris
RMS: Root mean square
SOL: Soleus
TA: Tibialis anterior
3DGA: 3-dimensional gait analyses
5MWT: 5-min walk test
